# The Use of an Acylhydrazone-Based Metal-Organic Framework in Solid-Contact Potassium-Selective Electrode for Water Analysis

**DOI:** 10.3390/ma15020579

**Published:** 2022-01-13

**Authors:** Paweł Kościelniak, Marek Dębosz, Marcin Wieczorek, Jan Migdalski, Monika Szufla, Dariusz Matoga, Jolanta Kochana

**Affiliations:** 1 Department of Analytical Chemistry, Faculty of Chemistry, Jagiellonian University, Gronostajowa 2, 30-387 Kraków, Poland; marek.debosz@doctoral.uj.edu.pl (M.D.); marcin.wieczorek@uj.edu.pl (M.W.); jolanta.kochana@uj.edu.pl (J.K.); 2 Department of Analytical Chemistry and Biochemistry, Faculty of Materials and Ceramics, AGH University of Science and Technology, A. Mickiewicza 30, 30-059 Kraków, Poland; migdal@agh.edu.pl; 3 Department of Inorganic Chemistry, Faculty of Chemistry, Jagiellonian University, Gronostajowa 2, 30-387 Kraków, Poland; monika.szufla@doctoral.uj.edu.pl (M.S.); dariusz.matoga@uj.edu.pl (D.M.)

**Keywords:** water analysis, potentiometry, ion-selective electrode, potassium, metal organic frameworks

## Abstract

A solid-contact ion-selective electrode was developed for detecting potassium in environmental water. Two versions of a stable cadmium acylhydrazone-based metal organic framework, i.e., JUK-13 and JUK-13_H2O, were used for the construction of the mediation layer. The potentiometric and electrochemical characterizations of the proposed electrodes were carried out. The implementation of the JUK-13_H2O interlayer is shown to improve the potentiometric response and stability of measured potential. The electrode exhibits a good Nernstian slope (56.30 mV/decade) in the concentration range from 10^−5^ to 10^−1^ mol L^−1^ with a detection limit of 2.1 µmol L^−1^. The long-term potential stability shows a small drift of 0.32 mV h^−1^ over 67 h. The electrode displays a good selectivity comparable to ion-selective electrodes with the same membrane. The K-JUK-13_H2O-ISE was successfully applied for the determination of potassium in three certified reference materials of environmental water with great precision (RSD < 3.00%) and accuracy (RE < 3.00%).

## 1. Introduction

Water is one of the most plentiful and essential compounds on the Earth and one of the most critical to life. Moreover, it is a good solvent for many substances, whose levels should be controlled. Water analysis is crucial in areas such as public health or environmental studies [1]. It covers the monitoring of such parameters such as physicochemical, biological and chemical properties. Water monitoring is also a determinant of a country’s development and indicates all the actions undertaken in order to reduce water pollution and improve water quality [2]. Investigations of water quality can be carried out using a lot of analytical techniques including classical ones, such as titrimetry or gravimetry, and modern ones, such as atomic absorption spectrometry (AAS), inductively coupled plasma-mass spectrometry (ICP-MS), photometry, or UV-Vis spectrophotometry [3]. The majority of them require sample pretreatment and do not comply with the principles of green chemistry [2].

Potassium occurs widely in the environment, including all natural waters. It is a necessary element for the normal functioning of a human body, as it maintains the normal osmotic pressure in all cells, and ensures proper functioning of the muscles and nerves. Moreover, it is vital for synthesizing proteins and metabolizing carbohydrates. An elevated level of potassium in the blood can lead to serious diseases such as kidney failure, heart attack, or diabetes. The need for controlling the potassium concentration in water and other sources is undoubtedly valuable not only from the diagnostic point of view, but also from the point of view of water quality assurance [4,5].

An analytical technique which is very simple to make, cheap, and fulfills the requirements of green chemistry is potentiometry. It is possible to determine the analytes directly at the sampling site with the use of portable analytical devices [6]. The potentiometric measurements are very often performed via in-line monitoring of water quality in various processes, without the use of special gases (as acetylene in FAAS, argon in ICP-OES or ICP-MS) or other special media or cooling agents for different spectrometers. Due to the use of several different electrodes sensitized for different analytes, it is possible to perform a multicomponent analysis at the same time in a very quick and easy way [7,8,9,10]. Moreover, the energy consumption in potentiometric measurements is much lower in comparison with other modern instrumental techniques [11]. The classical electrodes with inner filling solution are characterized by particularly good metrological parameters, fast response, and stability of measured potential. However, the presence of the inner filling solution hinders the miniaturization and the modification of the electrode shape. The development of solid-contact ion-selective electrodes allowed for the elimination of the above problems and kept the analytical parameters, i.e., stability and reproducibility of potential, at a similar level. The stabilization of the potential is offered due to the use of the ion-to-electron transducer layer playing the role of the inner filling solution in this type of electrodes. Numerous materials have been proposed as the solid contact, such as conducting polymers, carbon materials, nanomaterials, intercalation compounds, ionic liquids, or molecular redox couples [12,13]. However, the search for the perfect transducer material is still ongoing. The ideal material should be characterized by a reversible transition from ionic to electronic conduction, high exchange current density, stable chemical composition and possibly high hydrophobicity so that the formation of water between transducer and membrane interface is minimalized [14]. So far, the proposed materials, utilized as solid contact, do not meet all the mentioned requirements. Therefore, new materials are constantly proposed, the application of which would contribute to obtaining the desired metrological parameters of the solid contact (SC) electrodes. Recently, the use of organic metal frameworks as an ion-to-electron transducer was carried out and the presented results were encouraging [15].

Metal organic frameworks (MOFs) are an attractive sub-class of highly ordered and porous materials with two- or three-dimensional structures. MOFs are compounds composed of metal clusters (or ions) and bridging organic linkers as initially coined by Yaghi et al. [16] and further defined as IUPAC recommendations [17]. They possess numerous attractive properties including permanent porosity, abundant structures, high-surface area, good thermal stability, scalability and processability; all these properties contribute to the fact that MOFs found usage in a lot of different fields such as drug delivery, proton conduction, gas capture, separation, chemical and electrochemical sensing, catalysis, energy storage, etc. [18,19,20,21]. The possibility of the implementation of a multitude of strategies of MOFs’ synthesis can lead to materials with desirable properties for a given application [15]. Unfortunately, the majority of MOFs are electrical insulators which significantly limits the possibility of their use in electrochemistry, especially in potentiometry where they can play the role of a conductive ion-to-electron layer. However, some of the strategies for the improvement of the electric conductivity of MOFs are known. They include procedures such as the incorporation of ionic guest species, modification of the material with the use of redox-active or conductive compounds [22,23,24]. The use of MOFs in potentiometry is rare and only a few publications on this topic can be found in the literature. Mendecki et al. [15] presented the use of conductive MOFs as ion transducers in the solid contact potassium and nitrate selective electrodes. The proposed sensors exhibited perfect sensing properties, i.e., a near-Nernstian response and wide linear range. The utilized materials inhibited the formation of a water layer that led to the potential drift of only ca. 11 µV h^−^^1^. In Mahmoud’s work [25], Cu-MOF was utilized as an ionophore (modifier) in a carbon paste electrode for the detection of Al^3+^ ions in polluted water and pharmaceutical samples. The fabricated electrode was highly sensitive for Al^3+^ ions and allowed to obtain results with high precision and accuracy during the determination of the target ion in real samples.

The present work describes the preliminary studies of the implementation of two versions of a stable cadmium acylhydrazone-based metal organic framework, differing with guest molecules, as ion-to-electron transducers in solid contact ion-selective electrodes sensitive towards potassium ions. For the modification of a working glassy carbon electrode, we used a three-dimensional microporous MOF, {[Cd_2_(oba)_2_(tdih)_2_]·7H_2_O·6DMF}_n_ (JUK-13), built of 4,4′-oxybis(benzenedicarboxylate) (oba^2-^) and terephthalaldehyde di-isonicotinoylhydrazone (tdih) linkers (see Figure S1, Supplementary Material for linker formulas) [26]. Additionally, we used the same framework after exchanging DMF for water molecules, that is {[Cd_2_(oba)_2_(tdih)_2_]·13H_2_O}_n_ denoted as JUK-13_H2O [26]. Notably, the diacylhydrazone linker (tdih) decorates the pores of the MOF with the –CO-NH-N- groups that are potentially capable of chelating metal ions in solution through the formation of five-membered rings with O- and N-donor coordination bonds (Figure S1). We hypothesized that this ability can be advantageous for cation detection in aqueous solutions. Moreover, importantly, in the family of mixed-linker acylhydrazone-carboxylate MOFs known to date, JUK-13 stands out due to its high stability in water, confirmed previously by repeatable water vapor adsorption-desorption cycles and immersions in water, and due to its high N_2_ uptake and high pore volume in a two-dimensional channel system, as confirmed by crystal structure and adsorption isotherms [26]. The comparison of XRD patterns between pristine JUK-13 and JUK-13_H2O clearly indicates that JUK-13 undergoes a phase transition upon guest exchange (Figure S2). IR spectra demonstrate that this transition involves a rearrangement of the linker since characteristic bands corresponding to symmetric and asymmetric stretching of carboxylates considerably differ after guest exchange. Thermogravimetric analyses additionally confirm this exchange and demonstrate thermal stability of both materials up to approximately 300 °C (Figure S2). This phase transition, occurring upon immersion of JUK-13 in water, involves rearrangements of carboxylates and an exchange of guest molecules from DMF and water (present in the as-synthesized material) for water molecules only, which can be clearly observed by TGA and IR spectroscopy. The specific surface area of JUK-13 was determined by BET surface analysis to be 1010 m^2^ g^−1^ (Figure S3). In contrast, the analogue of JUK-13 functionalized by sulfonic groups (JUK-13-SO3H) easily degrades in liquid water, whereas it is stable under humid conditions [27]. The initial potentiometric characterizations were carried out for the electrodes modified by JUK-13 and JUK-13_H2O, and the latter was selected for further electrochemical studies. In order to verify the applicability of the proposed electrode, the sensor was utilized to analyze certified reference materials.

## 2. Materials and Methods

### 2.1. Reagents and Solutions

The following reagents were utilized for the ion-selective membrane preparation: potassium ionophore I (valinomycin) (Sigma Aldrich, Darmstadt, Germany), bis(2-ethylhexyl)sebacate (DOS) (Sigma Aldrich, Darmstadt, Germany), potassium tetrakis(pentafluorophenyl)borate (KTFAB, 97%) (Alfa Aesar, Ward Hill, MA, USA), high molecular weight poly(vinyl chloride) (PVC) and tetrahydrofuran (THF) (Sigma Aldrich, Darmstadt, Germany). Other chemicals, such as KCl, NaCl, LiCl, 1 mol L^−1^ HCl, ethanol, CaCl_2_ 2H_2_O, NH_4_Cl, and MgCl_2_ 6H_2_O were obtained from Merck Milipore. CH_3_COOLi 2H_2_O (Chempur, Piekary Śląskie, Poland) was also utilized. {[Cd_2_(oba)_2_(tdih)_2_]·7H_2_O·6DMF}_n_ (JUK-13) and its hydrated counterpart, {[Cd_2_(oba)_2_(tdih)_2_]·13H_2_O}_n_ (JUK-13_H2O) was synthesized according to literature procedure with a solvent-based approach [26]. Optical images of JUK-13 crystals in polarized light are shown in Supplementary Material (Figure S4). Notably, JUK-13 can also be obtained by an alternative solvent-free mechanochemical method.

Stock standard solutions of KCl (1 mol L^−^^1^), NaCl (1 mol L^−^^1^), LiCl (1 mol L^−^^1^), CaCl_2_ · 2H_2_O (1 mol L^−^^1^), NH_4_Cl (1 mol L^−^^1^), and MgCl_2_ ·6H_2_O (1 mol L^−^^1^) were prepared by dissolving adequate amounts of chloride salts in distilled water. Working solutions of potassium chloride, in the range of 10^−^^5^–10^−^^1^ mol L^−^^1^, were prepared immediately prior to the use in two solvents: distilled water (for the calibration procedure) and 10^−^^1^ mol L^−^^1^ lithium acetate (for the determination of potassium by calibration curve method). Ultrapure water (18.2 MΩ·cm) from an HLP 5 system (Hydrolab, Straszyn, Poland) was utilized throughout the work.

For the determination of selectivity coefficients, the solutions of each chloride salt, in the concentration range of 10^−^^3^–10^−^^1^ mol L^−^^1^, were prepared in distilled water. The 10^−^^1^ mol L^−^^1^ lithium acetate solution was prepared by dissolving an adequate amount of substance in distilled water and was utilized as an Ionic Strength Adjuster.

Certificate Reference Materials of Environmental Water, EnviroMAT Waste Water PlasmaCal, EnviroMAT Ground Water ES-H, EnviroMAT Drinking Water EP-H-1 (SCP SCIENCE, Baie D’Urfé, QC, Canada), were prepared by 50-fold dilution with lithium acetate solution.

### 2.2. Instrumentation

The potentiometric measurements were performed using a 16-channel Lawson Labs voltmeter equipped with the EMF Suite software (version 2.0.). Potentials were measured against Ag|AgCl|3 mol L^−^^1^ KCl|1 mol L^−^^1^ CH_3_COOLi reference electrode (Mineral, Warsaw, Poland). The bare or modified glassy carbon electrode (GCE, φ = 3 mm, BASi, West Lafayette, IN, USA) was utilized as a working electrode.

Electrochemical impedance spectroscopy (EIS) was executed using an Autolab Frequency Response Analyzer System (AUT20.FRA2-Autolab, Eco Chemie, B.V, The Netherlands). The measurement cell was composed of a tested electrode (the working electrode), Ag|AgCl wire (the reference electrode) and Pt wire (the counter electrode). The EIS measurements were carried out in 10^−^^1^ mol L^−^^1^ potassium chloride. The impedance spectra were recorded in a frequency range of 100 kHz–10 mHz, using a sinusoidal excitation signal with an amplitude of 10 mV. Before recording the spectra, the open-circuit potential (OCP) was measured.

All measurements were performed at room temperature.

### 2.3. Electrode Preparation

The surface of glassy carbon electrodes was polished with 0.3 µm Al_2_O_3_, rinsed and ultrasonicated in distilled water for 5 min. After ultrasonication, the glassy carbon electrodes were rinsed with distilled water, ethanol and again with distilled water. Then, the electrodes were air-dried.

The one component suspension containing 5 mg mL^−^^1^ of each Metal Organic Framework (JUK-13 and JUK-13_H2O) was prepared by weighing appropriate amounts of each MOF and mixing them with 0.5 mL of ethanol and then homogenizing with the use of an ultrasonic bath for 20 min.

The potassium selective membrane (K^+^-ISM) cocktail was composed of 1.06% (*w*/*w*) ionophore (Valinomycin), 0.34% (*w*/*w*) ion exchanger (KTFAB, 97%), 32.90% (*w*/*w*) polymer (PCV), and 65.80% (*w*/*w*) plasticizer (DOS). The components were dissolved in 1 mL of THF to produce a solution with 20% dry weight.

A total of 12.5 µL of MOF suspension was drop cast on the glassy carbon surface using an automatic pipette. The obtained MOF layer was allowed to dry in ambient conditions. Then, 40 µL of K^+^-ISM cocktail was deposited by drop-casting it onto the MOF layer in two consecutive steps (2 × 20 µL). The prepared solid-contact potassium-selective electrode was left overnight to allow the evaporation of the THF from the membranes. This procedure was utilized to prepare a series of electrodes. Once the THF solution evaporated, each electrode was conditioned in 10^−^^2^ mol L^−^^1^ KCl for at least 12 h. A calibration curve was received in the concentration range of 10^−5^–10^−^^1^ mol L^−^^1^.

### 2.4. Potentiometric Water Layer Test

The potentiometric water layer test was performed with fully conditioned solid-contact potassium-selective electrodes by recording the potential sequentially in 10^−^^1^ mol L^−^^1^ KCl, 10^−^^1^ mol L^−^^1^ NaCl and once again in 10^−^^1^ mol L^−^^1^ KCl. The solutions were stirred continuously during the measurements.

### 2.5. Selectivity Coefficients

The selectivity coefficients (K_I,J_^pot^) of the tested electrodes were determined by the separate solution method (SSM) [28]. The procedure was based on the measurements of the electromotive force (EMF) in the concentration range of 10^−^^3^–10^−^^1^ mol L^−^^1^ of chlorides of different cations. The values of selectivity coefficients were calculated by using the measured potentials in the solutions of main and interfering ion as parameters in the Nikolsky–Eisenman equation.

## 3. Results

### 3.1. Potentiometric Response and Potential Stability of MOF-Containing Potentiometric Potassium Sensor

**Figure 1:**
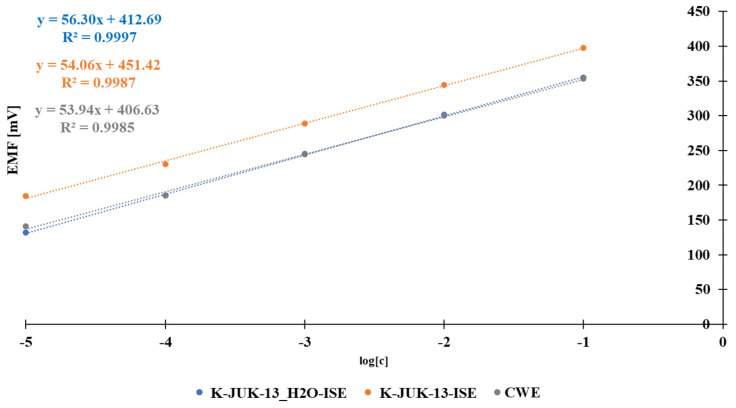
Calibration graphs for the constructed electrodes with MOFs as solid contact and CW electrode in the main ion concentration range 10^−5^–10^−1^ mol L^−1^.

After conditioning, the studied solid contact electrodes were calibrated in potassium chloride solutions with concentrations ranging from 10^−5^ to 10^−1^ mol L^−1^. For comparative purposes, the calibration was carried out for a coated wire electrode (CWE) as well, following the same scheme. The obtained calibration curves are shown in Figure 1. All tested electrodes exhibited good sensitivity to K^+^ ions in the same linear range. The CWE provided a linear response in the tested concentration range of K^+^ with a slope of 53.94 mV/decade. The use of JUK-13 as the ion-to-electron transducer does not have any impact on the response of a potassium selective membrane. This is confirmed by the obtained slope value of the calibration curve for the respective material. Moreover, the use of JUK-13_H2O caused the sensitivity of the electrode to increase by 2 mV/decade in comparison with the sensitivity of the CWE electrode.

**Figure 2:**
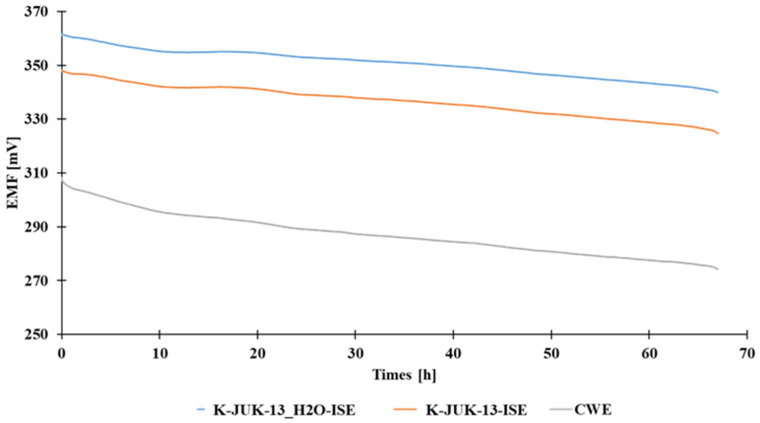
The change in potential of the tested electrodes with MOFs as ion-to-electron transducers and a coated wire electrode over time. The measurements were carried out in 10^−2^ mol L^−1^ KCl.

In order to verify the suitability of the proposed materials as the ion-to-electron transducers, the measurements of potential stability for the tested electrodes were performed in the following fashion. The potential readings were carried out in 10^−1^ mol L^−1^ potassium chloride solution for 67 h. During the measurements, the solution was stirred with a magnetic stirrer. For comparison, the stability measurements were also carried out for CWE. The obtained changes in the potential of the studied electrodes and a coated wire electrode over time are shown in Figure 2.

As shown in Figure 2, the use of the proposed MOFs as ion-to-electron transducers did not eliminate the potential drift. Only a slight reduction in the potential drift of solid contact electrodes in comparison with the CWE electrode is observed, which is confirmed by the data collected in Table 1.

The coated wire electrode exhibited the potential drift equal 0.51 mV h^−1^ and in the case of SC electrodes the values were lower by around 0.20 mV h^−1^. Among the tested versions of metal-organic frameworks, the use of JUK-13_H2O allowed for obtaining the lowest potential drift.

Based on the obtained sensitivities and potential drift values, the JUK-13_H2O was utilized in further studies.

### 3.2. Limit of Detection

**Figure 3:**
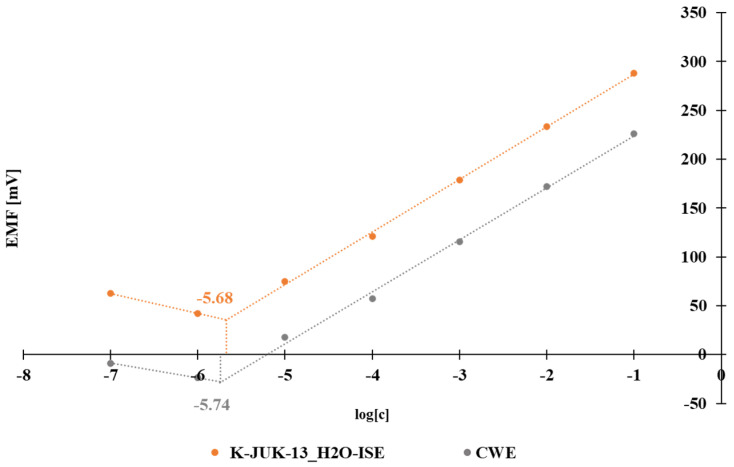
Determination of the limit of detection for K-MOF-ISE and CWE.

In order to determine the limit of the detection (LOD) of the studied SC and CW electrodes, the calibration measurements in the concentration range 10^−7^ to 10^−1^ mol L^−1^ were carried out. The LOD was calculated as the intersection of two lines as shown in Figure 3. For SC-type and CW-type electrodes, the LOD equals 2.1 × 10^−6^ mol L^−1^ and 1.8 × 10^−6^ mol L^−1^ K^+^, respectively. The utilized MOF has no impact on the LOD value.

### 3.3. Selectivity Coefficients

The potentiometric selectivity coefficients of the tested electrodes, with and without the MOF, using the chloride salts of different cations, were determined by the separate solution method. The obtained values are shown in Table 2. In order to evaluate the obtained selectivity coefficients, the data obtained for a potassium selective electrode with multiwalled carbon nanotubes modified with octadecylamine are presented in Table 2. Each of the tested electrode groups utilized the ion-selective membrane with the same composition.

The results proved that the CWE and SC electrodes exhibit good selectivity towards their primary ion. The determined selectivity coefficients were similar to the values reported in the literature [29,30] and they are close to the coefficients obtained for an SC-type electrode with OD-MWCNTs [7]. The selectivity coefficients should, by definition, be dependent on the composition of the utilized ion-selective membrane and not on the type of the utilized ion-to-electron transducer. The obtained results confirmed this rule. Among the interfering ions, the most notable interfering cations for the tested electrode are ammonium ions, while sodium ions show the weakest influence on the electrode response.

### 3.4. Water Layer Test

The formation of an undesirable water layer between the polymeric ion-selective membrane and ion-to-electron transducer can lead to potential instability of solid contact electrode and cause mechanical failure. The composition of this layer varies upon sample changes leading to many unfavorable processes, for instance, sensitivity to the CO_2_, slow equilibrium process or longer time of response.

**Figure 4:**
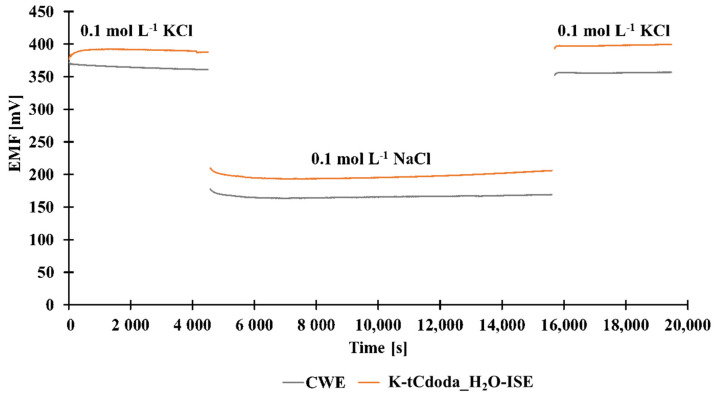
Water layer test of K-JUK-13_H2O-ISE and CWE.

The water layer test was carried out according to the procedure suggested by Fibbioli et al. [31]. Before the test, the electrodes, i.e., K-JUK-13_H2O-ISE (SC-ISE) and CWE, were conditioned in 10^−1^ mol L^−1^ of primary ion solution. First, the potential of the electrodes was recorded in 10^−1^ mol L^−1^ KCl for 1 h, then in a solution of the interfering ion (10^−1^ mol L^−1^ NaCl). After 3 h, the interfering ion solution was replaced by a primary ion solution. The obtained results are shown in Figure 4.

The obtained results suggest that there is a positive drift of the potential when the solution of the primary ion is substituted with the solution of the interfering ion for both types of electrodes. It is caused by the leaching of primary ions from an ion-selective membrane contributing to the growth of the potassium ion concentration in the near membrane solution layer and to the increase in the electrodes’ potential. After placing the electrodes back to the primary ion solution, the potential of the tested electrodes returned to the values close to the initial ones.

The data indicate that the use of glassy carbon electrodes with the MOF as a solid contact leads to a positive result of the water layer test similarly as in the case of the coated wire electrode.

The proposed electrodes can work for one month or longer after the first calibration, but their response is poorer and the linear range is narrower each day; this is caused by the leaching of the components from the membrane. For analytical studies, the electrodes were maximally utilized for one week after the first calibration, when the difference in sensitivity did not exceed 3%.

### 3.5. Electrochemical Impedance Spectroscopy

**Figure 5:**
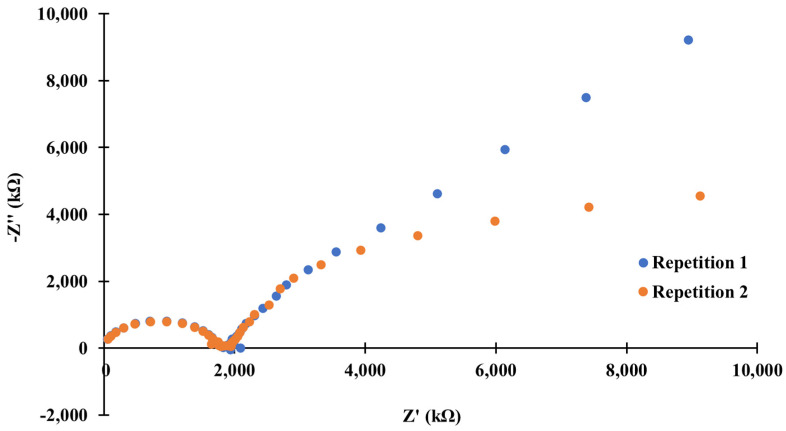
Impedance spectra of the studied solid-contact potassium-selective electrode with JUK-13_H2O as an ion-to-electron transducer.

EIS measurements aimed to study the function of the MOF as a transducer layer and to verify whether the tested material possesses good electrical and ionic conductivity or/and sufficiently high redox capacitance providing the stability of measured potential during the flow of a little electric charge through the system. The spectra were measured in 10^−1^ mol L^−1^ potassium chloride solution. The impedance spectra of the studied electrode with the MOF as the transducer layer are shown in Figure 5.

The obtained spectra possess a semi-circle in the high-frequency range corresponding to the membrane resistance connected parallelly with electrical capacitance. However, in the case of the low-frequency range, there is a branch indicating the presence of a charge transfer resistance between ion-to-electron transducer, in this case, the MOF or electrode substrate-glassy carbon, and an ionically conducting membrane. Since the selected metal organic framework is insoluble in typical solvents, it forms a colloidal suspension, which leads to the fact that the shape of the recorded spectra can be affected by the intergranular resistance. The yielded spectra correspond in their shape to the typical spectra recorded for coated wire electrodes. This indicates that blocking of the charge flow between the mediation layer and the electrode substrate or an irreversible redox reaction is taking place. This was also confirmed by the fact that the measured open circuit potential (OCP) changed its value between the first and the second repetition from 0.26 V to 0.38 V. As a result of the redox reaction, the change in the ratio of the reduced to the oxidized form in the ion-to-electron transducer occurred, contributing to the change in the measured potential. On the basis of the obtained results, it can be concluded that the proposed material exhibits poor ionic and electrical conductivity and possesses low redox capacitance.

### 3.6. Analytical Application

The proposed solid-contact potassium-selective electrode was verified by determining the content of potassium in three certified reference materials (CRMs) of environmental waters: wastewater, drinking water and ground water. The calibration curve method was utilized as a calibration method. Each CRM sample was diluted 50-fold and analyzed six times. Along with the values of relative errors (RE (%)), confidence intervals (*n* = 6, α = 0.05) were calculated in order to compare the results with certificate values. As an ionic strength adjuster, lithium acetate solution (0.1 mol L^−1^) was utilized. The results are shown in Table 3.

The obtained results are satisfying in terms of both accuracy and precision. The values of relative error (RE (%)) and relative standard deviation (RSD (%)) do not exceed 3.00%, which points to a very good precision and accuracy of the results. The values of the considered parameters are favorable from an analytical point of view due to the use of an ionic strength adjuster which has a significant impact on the repeatability of recorded signals.

## 4. Conclusions

This work demonstrates the first use of acylhydrazone-based MOFs as modifiers of working electrodes for potentiometric sensing in aqueous solutions. The properties of the proposed MOF enable its use for potentiometric detection. The sensor exhibited good performance characteristics, including a near-Nernstian response, wide linear range (from 10^−5^ to 10^−1^ mol L^−1^), good selectivity, and good potential stability in comparison with the CW-type electrode. Moreover, the proposed sensor was successfully applied for K^+^ determination in certified reference materials of different water samples with very good accuracy and precision. Naturally, future efforts should focus on rare electrically conductive MOFs as the coexistence of both porosity and high conductivity are desirable for more efficient ion-to-electron transducers. The literature reports on the use of this class of materials in potentiometric measurements indicate that this topic is worth pursuing.

## Figures and Tables

**Table 1:** Long-term potential stability of the studied solid contact and coated wire electrodes.

Time (h)	Potential (mV)		
	K-JUK-13_H2O-ISE	K-JUK-13-ISE	CWE
0.00	361.58	348.27	308.28
67.00	339.90	324.70	274.21
Drift (mV h^−1^)	0.32	0.35	0.51

**Table 2:** Comparison of the potentiometric selectivity coefficients for proposed K-JUK-13_H2O-ISE, coated wire electrode (CWE) and K-OD-MWCNTs-ISE (with the same ion-selective membrane) (*n* = 3, α = 0.05).

Interferent	K_K.J_^pot^		
	K-JUK-13_H2O-ISE	CWE	K-OD-MWCNTs-ISE
Li^+^	−3.90 ± 0.09	−4.19 ± 0.43	−3.69 ± 0.66
Ca^2+^	−3.84 ± 0.46	−3.71 ± 0.41	−4.18 ± 0.30
Mg^2+^	−4.99 ± 0.20	−4.97 ± 0.22	−3.99 ± 0.76
NH_4_^+^	−2.21 ± 0.42	−2.61 ± 1.48	−1.64 ± 0.71
Na^+^	−4.23 ± 0.13	−4.43 ± 0.01	−3.89 ± 0.27
H^+^	−4.00 ± 0.13	−4.02 ± 0.01	−4.73 ± 1.91

**Table 3:** Results of potassium determination in certified reference materials using the calibration curve method (C_o_ and C_x_—certified for CRM and found concentrations respectively; RE—relative error; RSD—relative standard deviation) (*n* = 6, α = 0.05).

Sample	C_0_ (mmol L^−1^)	C_x_ (mmol L^−1^)	RE (%)	RSD (%)
Waste Water EU-H-3	1.02	1.05 ± 0.02	2.9	2.4
Drinking Water EP-H-1	0.33	0.33 ± 0.01	1.9	1.7
Ground Water ES-H	0.074	0.073 ± 0.002	−1.4	2.8

## Data Availability

Data available in a publicly accessible repository. The data presented in this study are openly available in Jagiellonian University Repository at DOI: 10.26106/cjky-4381.

## Supplementary Materials

The following supporting information can be downloaded at: https://www.mdpi.com/article/10.3390/ma15020579/s1, Supplementary materials contain additional measurement data (Figures S1−S4) and descriptions for the MOF’s: JUK-13_H2O and JUK-13_H2O. Figure S1: (Top) Scheme of synthetic procedure for {[Cd2(oba)2(tdih)2]·guests}n (JUK-13); (Bottom) Microporous structure of JUK-13; Figure S2: Comparison of JUK-13 and JUK-13_H2O MOFs: a) PXRD patterns indicate phase transition upon guest exchange in water. b) IR spectra demonstrate that the phase transition involves rearrangements of carboxylates. c) Thermogravimetric analyses confirm the presence of various guest molecules in JUK-13 and JUK-13_H2O and the stability of both materials up to ca. 300 OC upon pore evacuation; Figure S3: Physisorption isotherm of N2 (77 K) for JUK-13; Figure S4: Optical images of JUK-13 crystals in a polarized light: dark field (top) and bright field (bottom).
