# Supplementary material for: The Use of an Acylhydrazone-Based Metal-Organic Framework in Solid-Contact Potassium-Selective Electrode for Water Analysis
Source: Materials (Basel). 2022 Jan 13;15(2):579. doi: 10.3390/ma15020579 (PMC8781722; doi:10.3390/ma15020579)
Supplement: Supplementary file 1 [file materials-15-00579-s001.zip › materials-1488207-supplementary.pdf]

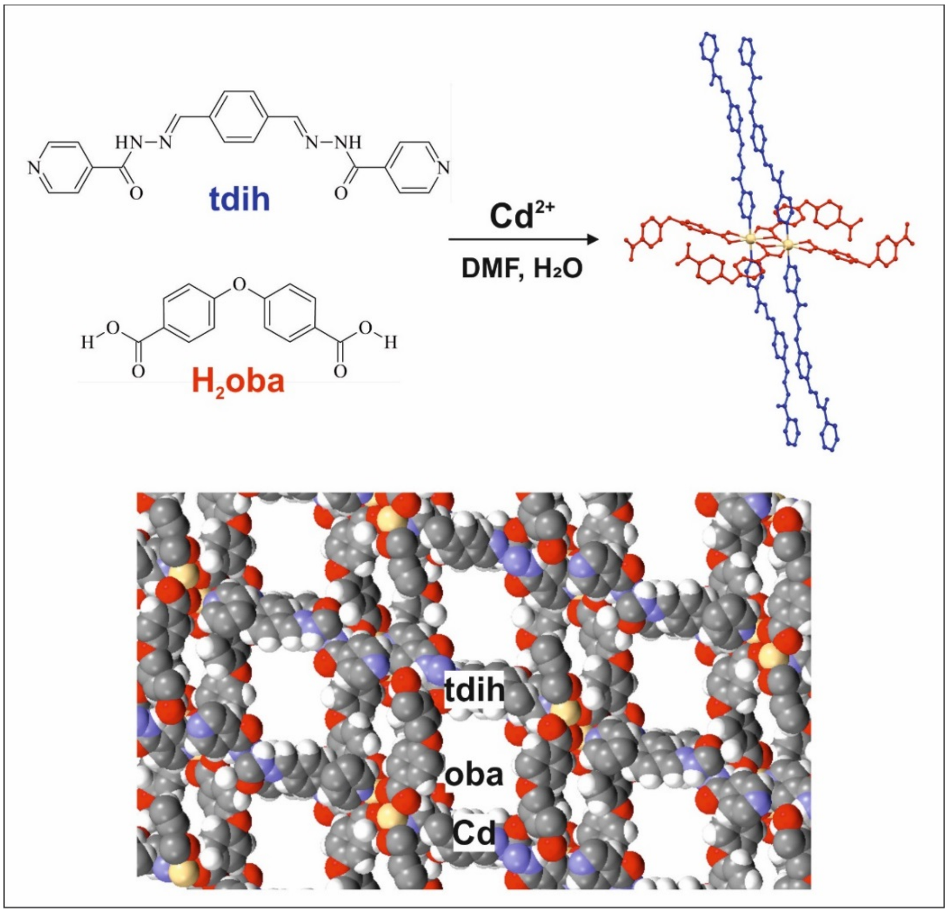

Figure S1. (Top) Scheme of synthetic procedure for $\{[Cd_2(oba)_2(tdih)_2]\cdot guests\}_n$ (JUK-13); blue – a diacylhydrazone linker (tdih), red –a dicarboxylate linker (oba^{2-}), yellow – cadmium ion; (Bottom) Microporous structure of JUK-13: one-dimensional channels in a spacefill representation (according to Zeo++ calculations window size and maximum pore diameter are: 6.9 Å and 9.1 Å, respectively); Cd-yellow, C-grey, O-red, N-blue, hydrogen-light grey.

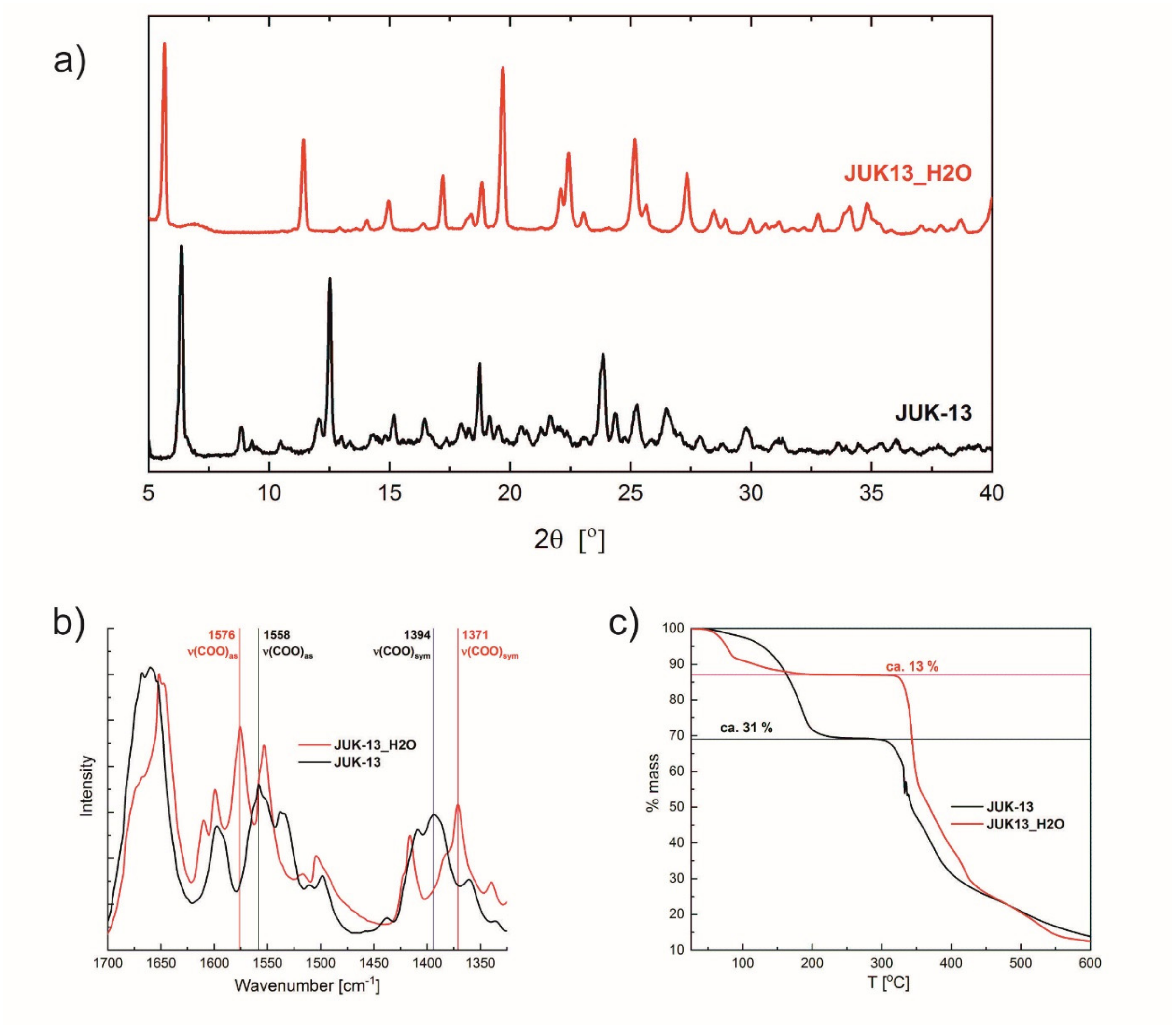

Figure S2. Comparison of JUK-13 and JUK-13_H2O MOFs: a) PXRD patterns indicate phase transition upon guest exchange in water. b) IR spectra demonstrate that the phase transition involves rearrangements of carboxylates. c) Thermogravimetric analyses confirm the presence of various guest molecules in JUK-13 and JUK-13_H2O and the stability of both materials up to ca. 300 °C upon pore evacuation.

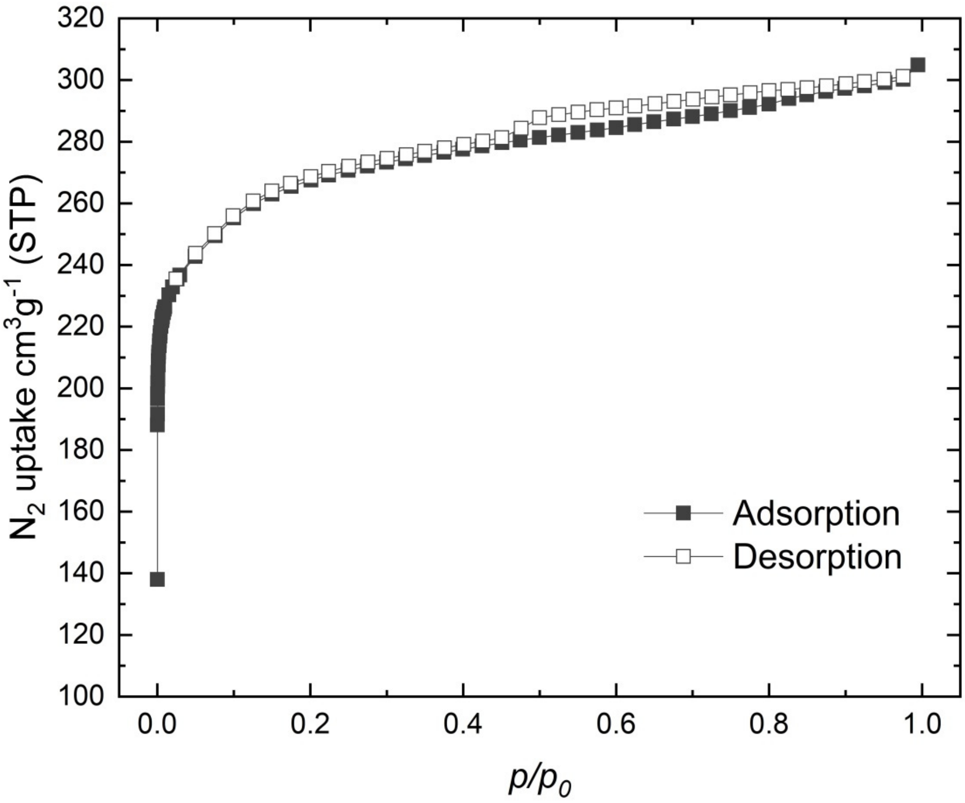

Figure S3. Physisorption isotherm of N_2 (77 K) for JUK-13 synthesized in solution and activated as described below.

BET surface area: 1010 $m^2\,g^{-1}$

Nitrogen physisorption measurement was performed on an Autosorb iQ-C-XR-XR EPDM apparatus (Quantachrome Instrument) at 77 K (achieved by liquid nitrogen bath). Prior to the sorption measurements, samples were immersed in methanol for 24 h. During this time methanol was replaced three times by a fresh portion. After that, sample was activated at 100 °C under vacuum for 8 h.

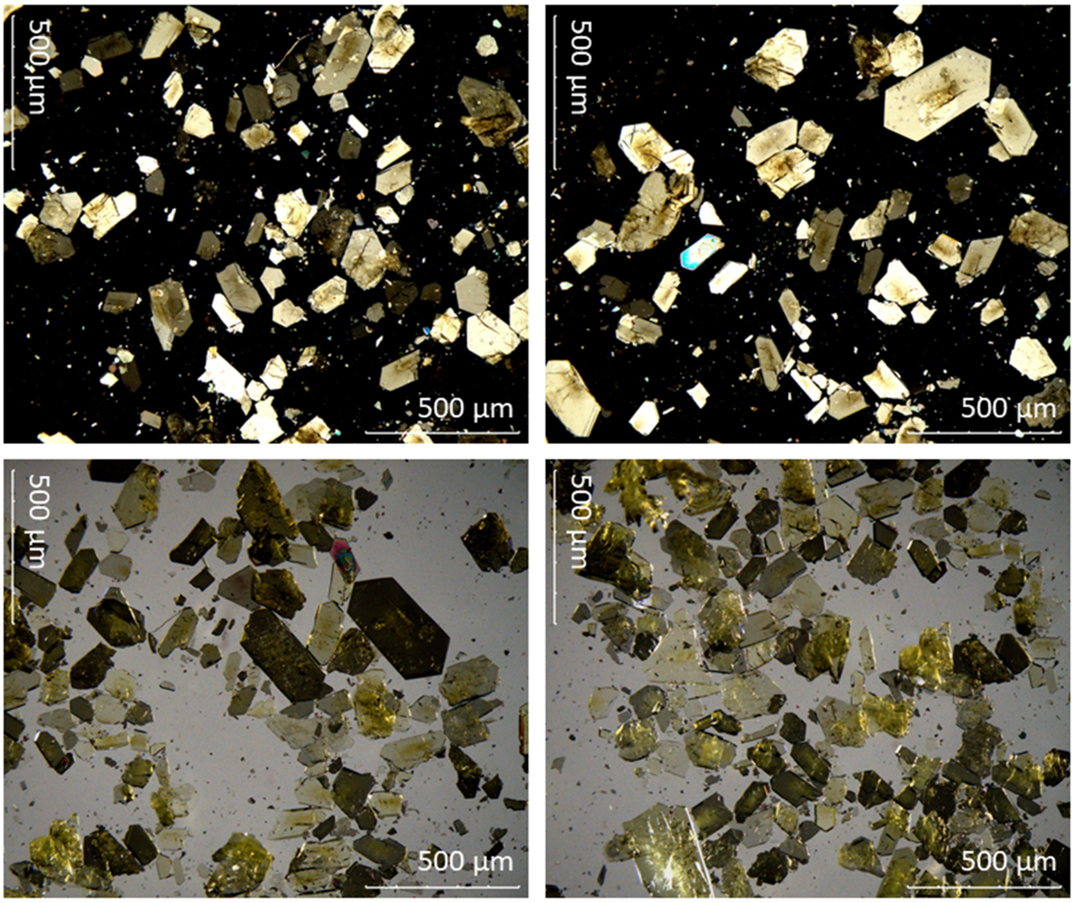

Figure S4. Optical images of JUK-13 crystals in a polarized light: dark field (top) and bright field (bottom).

Scanning electron microscopy (SEM) images of JUK-13 samples obtained by one-pot mechanochemical method from $Cd(OH)_2$ precursor are presented in Cryst. Growth Des. 2019, 19, 7160–7169, ESI, Figure S5. Crystallite sizes depend on a synthetic method: in solution they reach sizes on the order of 10^2 µm (Figure 4), while the mechanochemical approach results in sizes on the order of microns.
